# Innate Pattern Recognition and Categorization in a Jumping Spider

**DOI:** 10.1371/journal.pone.0097819

**Published:** 2014-06-03

**Authors:** Yinnon Dolev, Ximena J. Nelson

**Affiliations:** School of Biological Sciences, University of Canterbury, Christchurch, New Zealand; University of Sussex, United Kingdom

## Abstract

The East African jumping spider *Evarcha culicivora* feeds indirectly on vertebrate blood by preferentially preying upon blood-fed *Anopheles* mosquitoes, the vectors of human malaria^1^, using the distinct resting posture and engorged abdomen characteristic of these specific prey as key elements for their recognition. To understand perceptual categorization of objects by these spiders, we investigated their predatory behavior toward different digital stimuli - abstract ‘stick figure’ representations of *Anopheles* constructed solely by known key identification elements, disarranged versions of these, as well as non-prey items and detailed images of alternative prey. We hypothesized that the abstract images representing *Anopheles* would be perceived as potential prey, and would be preferred to those of non-preferred prey. Spiders perceived the abstract stick figures of *Anopheles* specifically as their preferred prey, attacking them significantly more often than non-preferred prey, even when the comprising elements of the *Anopheles* stick figures were disarranged and disconnected from each other. However, if the relative angles between the elements of the disconnected stick figures of *Anopheles* were altered, the otherwise identical set of elements was no longer perceived as prey. These data show that *E. culicivora* is capable of making discriminations based on abstract concepts, such as the hypothetical angle formed by discontinuous elements. It is this inter-element angle rather than resting posture that is important for correct identification of *Anopheles*. Our results provide a glimpse of the underlying processes of object recognition in animals with minute brains, and suggest that these spiders use a local processing approach for object recognition, rather than a holistic or global approach. This study provides an excellent basis for a comparative analysis on feature extraction and detection by animals as diverse as bees and mammals.

## Introduction

Object recognition is the ability to perceive the physical properties (such as shape, color and texture) of an object and apply semantic/cognitive attributes to the object [1], such as an understanding of its use, or classification of the object as prey, predator or irrelevant. The process leading to recognition is typically, though not exclusively, viewed as a bottom-up hierarchy in which information is processed sequentially with increasing complexity. In vertebrates, the idea is that lower-level cortical processors, such as the primary visual cortex, process the basic object components such as color, depth and form, while higher-level cortical processors, such as the inferotemporal cortex in humans, are ultimately responsible for recognition [2]. Historically, perhaps one of the best-known attempts at explaining perception and recognition is that of Gestalt psychology.

The central tenet of Gestalt psychology is that the whole differs from the sum of its parts. The theoretical framework underlying Gestalt ideas is holism, which states that systems and their properties should be viewed as wholes, not as collections of parts [3]. This contrasts with earlier structuralist hypotheses, which state that perceptions can be derived by identifying the elementary parts [4]–[6]. Modern research into visual processing has changed its focus from gestaltism vs. structuralism to global vs. local processing [6], [7], with an expanded focus from the psychological processes of perception to include physiological processes [8]. The global processing framework results in the notion that an object is recognized only when its elements form the whole image, while the local processing framework requires the identification of correct elements, points and edges, but not necessarily the image as a whole. This distinction also suggests potential differences in the neurobiological processes underlying object recognition [8].

For a predator that relies on vision, visual ability to classify an object as predator or prey will be under strong selection. However, the extent to which visual predators further classify items can vary considerably. Some predators make rapid decisions and do minimal classifying of prey into particular types, relying instead on key features, such as seeing an object of a specific size range moving in a specific orientation, as identifiers of prey [9]–[12]. Examples of this approach can be found among amphibians [12], [13] and mantises [14], which adopt remarkably similar approaches despite possessing very different nervous systems. Many jumping spiders (Salticidae) also rapidly categorize objects as prey or non-prey based on only a few key features [15]–[18]. However, it is also amongst the salticids that some of the most precise prey identification and prey preference behaviors among animals is found.

An extreme case of such preference is that of *Evarcha culicivora*. Uniquely, this East African salticid feeds indirectly on vertebrate blood by selectively preying upon female mosquitoes (particularly *Anopheles*, famous as the vectors of malaria) that have recently fed on blood. These spiders are capable of using vision alone to discriminate between their preferred prey, blood-fed female *Anopheles,* and similar looking male *Anopheles*, female *Anopheles* that have not fed on blood, non-anopheline mosquitoes, as well as various similar-sized non-mosquito prey [19]–[21]. These experiments have also shown that for correct identification *E. culicivora* uses a complex non-linear process involving specific elements of the prey, including an engorged abdomen, resting posture and antennae [20], [22].

Like other salticids, *E. culicivora* has exceptional eyesight, which is used to locate, stalk and finally pounce on its prey [23]. Salticids have large forward-facing principal eyes that are specialized for high resolution vision but within a very narrow (ca. <5°) field of view [24]–[28] which is compensated for with complex movements that scan up to ca. 28° to either side of the body axis [27]. Additionally, salticids have three pairs of motion-sensitive secondary eyes with wide fields of view and which collectively encompass up to 360° [29]–[32].

*E. culicivora*’s unique dietary preferences, which can be expressed using vision as the sole sensory modality for prey classification [20], [22], make this species an excellent subject for the study of recognition and classification of prey. Here we presented the spiders with abstract representations of potential prey (‘stick figures’) differing in their level of simplicity to determine whether predatory behavior and prey classification was elicited by biologically unrealistic prey containing only key elements (local processing). Stimuli included stick figures of *Anopheles* mosquitoes in their resting posture, as well as non-prey items and alternative prey items. We used single-choice predatory behavior experiments to determine whether or not *E. culicivora* ‘viewed’ abstract representations of prey as potential prey, and two-choice predatory behavior experiments to test for specific preference between stimuli. Due to *E. culicivora*’s known ability to discern specific elements of prey, we predicted that these specialized visual hunters would stalk and pounce on abstract representations of prey. We also predicted that *E. culicivora* would choose simplified representations of its preferred prey over realistic images of alternative non-preferred prey, showing that it categorizes these images as its preferred prey item.

## Results

### a. Do Jumping Spiders View Abstract Images of Prey Elements as Prey?

A total of 195 successful sessions were run in the single-choice predatory behaviour experiment: 85 with adult females, 50 with adult males and 60 with juvenile spiders. When spiders initiated stalking behavior, this almost always resulted in pouncing on the abstract prey (Table S1). The type of stimulus had no effect on whether the spiders noticed it (χ^2^ = 6.71, df = 6, p = 0.349, Table S2). Stimulus type did affect the propensity to stalk the prey once it was noticed (χ^2^ = 37.87, df = 6, p<0.001), but did not affect the amount of time it took the spiders to ‘decide’ to stalk the prey (time between the spider first noticing the stimulus and initiation of stalking behavior; χ^2^ = 3.928, df = 6; p = 0.686, Table S2). Once stalking was initiated, stimulus type had no effect on the propensity to pounce (χ^2^ = 4, df = 6, p = 0.677, Table S2). We therefore considered stalking to be a true sign of predatory behavior by the spiders. The spiders stalked the abstract images of mosquitoes (stimuli 1, 2, 3 and 4) significantly more often than the images of non-prey items (stimuli 6 and 7; Table 1). However, while the image of the fly (stimulus 5) was stalked significantly more often than the altered, disarranged abstract image of the blood-fed mosquito (stimulus 7), it wasn’t stalked more often than the image of the circle (stimulus 6).

**Table 1:** Responses and statistical comparisons of the spiders to the different stimuli.

Stimulus	1	2	3	4	5	6	7
N	27	28	27	30	29	27	27
Stalk (%)	74	64	74	77	56	33	24
**1**	-	0.508	1	1	0.267	<0.001	<0.001
**2**	-	-	0.549	0.549	0.774	<0.05	<0.005
**3**	-	-	-	1	0.267	<0.05	<0.001
**4**	-	-	-	-	0.302	<0.005	<0.001
**5**	-	-	-	-	-	0.118	<0.05
**6**	-	-	-	-	-	-	0.219

**Figure 1:**
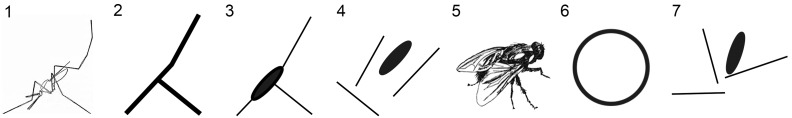
Images (and numbering as referred to in text) used as stimuli in both experiments. Images 1–4 are based on Anopheles mosquitoes. 1 is based on [60]. Image 4 is a disarranged version of image 3. Image 8 is a disarranged version of image 6. Image 7 is based on image 4 where the angles of the various elements have been altered.

Upper section of the table contains the number of spiders that noticed each stimulus (N) and the percentage of stalking instances. The bottom section of the table contains the crosswise comparisons of the stalking responses to the different stimuli, using McNemar tests with a binomial distribution. See Figure 1 for stimulus images. Note all image sizes are equivalent, see Table 3.

GLMs on the propensity to stalk showed significant main effects of stimulus type (χ^2^ = 22.315, df = 6, p<0.005) and spider sex (χ^2^ = 7.413, df = 2, p<0.05), but not their interaction (χ^2^ = 9.270, df = 11, p = 0.597). The effects of the relative contrast of the stimuli or its interaction with spider sex were also not significant (respectively, χ^2^ = 0.039, df = 1, p = 0.843; χ^2^ = 0.431, df = 2, p = 0.806). Females and juveniles were more prone to stalk stimuli (56.5% of 85 and 70% of 60 respectively) than males (38% of 50; females vs male: U = 1732.5, p<0.05; juveniles vs males: U = 1020, p<0.001, Mann-Whitney U test, Data, Tables S9, S11), while there was no significant difference between females and juveniles (U = 2205, p = 0.099, Mann-Whitney U test, Data, Table S11). Similarly, stimulus type had a significant effect on the propensity of females and juveniles to stalk (respectively,:: Cochran’s Q = 14.195, p<0.05; Cochran’s Q = 14.261, p<0.01, Data, Tables S3, S4, S7, S8) but not on that of the males (Cochran’s Q = 5.636, p = 0.465, Data, Tables S5, S6). While there were no significant differences in how often the different sexes noticed different stimuli (χ^2^ = 5.762, df = 2, p = 0.056, Kruskal-Wallis test, Data, Table S10), there were significant differences in the distances at which the they noticed the stimuli (χ^2^ = 14.021, df = 2, p<0.005, Kruskal-Wallis test, Data, Tables S10), with the females noticing the stimuli from significantly further away than males or juveniles (respectively, U = 1471, p<0.005; U = 1751, p<0.0.005, Mann-Whitney U tests, Data, Table S11). There were also significant differences between the sexes in their propensity to pounce once stalking was initiated (χ2 = 10.461, p<0.01, Kruskal-Wallis test, Data, Tables S9, S10), with the males less prone to pounce on prey than either females or juveniles (respectively: U = 306.5, p<0.01; U = 267, p<0.01, Mann-Whitney U tests, Data, Table S11). See Data, Tables S1–S11 for the full datasets.

### b. Do Jumping Spiders View Abstract Images as their Preferred Prey?

A total of 123 successful sessions were run in the two-choice predatory behaviour experiments, 61 with females, 34 with males and 28 with juveniles. Spiders never exhibited a side bias (experiments 1 through 5, respectively: p = 0.23; p = 0.83; p = 0.35; p = 0.54; p = 0.54, Binomial test). When given a choice between abstract representations of their preferred prey and a realistic image of non-preferred prey (a house fly), *E. culicivora* chose the preferred prey significantly more often (experiments 1 and 2 respectively, p<0.001; p<0.01, Binomial test, Table 2). Spiders also chose a disarranged abstract representation of their preferred prey significantly more often than they chose a realistic image of non-preferred prey (experiment 3, p<0.05, Binomial test, Table 2), or a disarranged non-prey item (experiment 5, p<0.05, Binomial test, Table 2). However, spiders showed no preference when presented with an abstract representation of their preferred prey and a disarranged version of that same image (experiment 4, p = 0.84, Binomial test, Table 2).

**Table 2:** Results of two-choice predatory behavior experiment stimulus pairs. Note all stimulus sizes are equivalent, see Table 3.

Experiment	N	Image 1	Image 2	Chose Image 2	p
1	22	2	5	9%	<0.001
2	28	3	5	25%	<0.05
3	28	4	5	29%	<0.05
4	24	3	4	46%	0.84
5	21	4	8	19%	<0.05

Pairs of images used in the two-choice predatory behavior experiments, percentage of pounce choices for the second image, and results of Binomial tests. See Figure 1 for images.

## Discussion

This study shows that for *E. culicivora,* discrimination and categorization can be achieved using only visual representations of the basic elements of its preferred prey. By using stick figure drawings of their preferred prey – *Anopheles* mosquitoes, we have created stimuli constructed only of key elements of their prey that have been found to be important for recognition [20], [22]. As hypothesized, we have shown that not only do these spiders view these stimuli as potential prey (by initiating predatory behavior), but they also prefer these abstract images of prey to detailed images of alternative non-preferred prey. These results show that the various elements that have been found to be necessary for prey discrimination in previous studies are [20], [22] also sufficient for recognition. This was the case regardless of whether or not the spiders had encountered their preferred prey before. Our controls have ruled out external cues, such as side preference, number of elements of the stimulus, and the relative contrast of the stimuli. Interestingly, the propensity to pounce was not affected by the different stimuli, and was seen in almost all cases where stalking was initiated. It would seem that the decision to pounce relies on other cues not singled out in this study, or, perhaps more likely, that pouncing is a follow-up behavior akin to a ‘fixed action pattern’.

Our confidence in these results is strengthened by the behavior of the naïve juveniles in the single-choice predatory behavior experiment. When hunting *Anopheles*, but no other type of prey, juveniles of *E. culicivora* perform an innate prey-specific predatory behavior involving a detour to approach the prey from behind [33]. This detouring approach to the prey was evident in 57% of the trials involving a stimulus representing an *Anopheles* (stimuli 1–4; N = 31; stimulus 1 (detours/attacks): 7/11; stimulus 2: 4/9; stimulus 3: 6/10; stimulus 4: 1/1) with juveniles, but only once with the fly stimulus (stimulus 5; N = 8) and never with the circle stimulus (stimulus 6; N = 3). Despite these small sample sizes, it is apparent that they recognize the stick-figure stimuli specifically as *Anopheles* mosquitoes.

The low level categorization of the abstract stimuli into prey and non-prey items is also seen in other invertebrates such as the praying mantis, where basic features of the stimuli, including size and speed, are the main cues [14], [34]. However, *E. culicivora*’s discriminations use much finer details of an image, such of the size and shape of mosquito antennae, when making decisions regarding preference [20], [22], and thus require a considerably higher level of feature detection. The most notable instance of such discrimination in this study was the ability of the spiders to discriminate between the two disarranged stimuli in the single-choice predatory behavior experiment, where the only difference between the stimuli were the relative angles between the elements and yet one was categorized as prey, while the other was not. Nelson and Jackson [20], [22] have shown that the resting posture of a mosquito is an important cue for recognition. Our findings fine-tune those conclusions by suggesting that it is not the angle of the body compared to a surface or horizon, but rather the relative angles between the body elements that is crucial for recognition.

Discrimination of orientation has been shown in honeybees (*Apis mellifera*), which can distinguish different orientations even when these are produced through illusory contours [35] and without clear edge detection [36]. Horridge [37], [38] proposed that the generalization ability of the honeybee uses different parameters of an image to form local cues. These discrimination mechanisms were based on physical aspects of an image, but Avargues-Weber et al. [39], [40] demonstrated that honeybees are even capable of abstract concepts such as above-below and left-right. Unlike in the bee studies, we used unlearned stimuli and untrained animals, and show that *E. culicivora* is capable of discrimination using a significantly more complex abstract concept - angles between disconnected elements.

One way of achieving such discrimination ability is by storing the ‘correct’ orientation of the various elements and comparing each element to stored memory. However, the spiders occasionally pounced upon the stimulus while standing on the sides or the ceiling of the starting chamber (analogous to behavior common in a natural setting, XJN pers. obs.), suggesting that orientation effects do not play a role in these decisions. While it is tempting to consider this type of object consistency in recognition to be superior to that seen in human recognition of faces (where face recognition is degraded significantly more than other objects when viewed upside-down [41]–[44]), there is an inherent difference between the two - faces often have a prototypical orientation, while in the spider’s natural three-dimensional environment prey is often viewed from different orientations.

An alternative mechanism of achieving the discrimination ability seen in this study is by ‘calculating’ the relative difference of the angles and comparing that to stored angles that represent prey. While discrimination of orientation has been well studied in vertebrates and invertebrates [45]–[49], relative angle discrimination in non-human animals remains largely unstudied. In humans, however, this ability has been well studied (e.g., [50]–[52]) and there is some evidence for a neural mechanism that encodes angles in humans [53], as well as in macaques [54] and cats [55].

Our results demonstrate that *E. culicivora* not only categorizes the simplified abstract stimuli as prey, but recognizes them as its preferred prey, exhibiting higher level categorization or within-category discrimination. This was the case even for the disarranged version of the blood-fed *Anopheles*, a capability not dissimilar to that of humans with visual expertise when viewing fragmented images of cars or faces [56], although in our case the images were abstract and dispersed rather than fragmented. *E. culicivora* not showing any preference between the blood-fed *Anopheles* stimulus and its disarranged version was perhaps the most surprising finding of this study. While it is possible that *E. culicivora’s* response to the image of the disarranged *Anopheles* was due to its resemblance to some other unknown prey rather than *Anopheles*, this is unlikely as the dietary preferences of these spiders has been well studied [19], [20], [22], [33]. We should note that experiments using stimuli 4 and 7 were both run at a later date. While this too might have affected the results, this also seems unlikely, as the laboratory conditions were constant and the spiders were healthy. Another alternative explanation is that the specific arrangement of the elements of the disarranged *Anopheles* exploits a sensory bias in the *E. culicivora’s* visual pathways, while the altered version of this stimulus does not. Regrettably, we could not test the spider’s responses to other alternative arrangements of these stimuli. Nonetheless, either through a sensory bias in the visual pathways, or by higher level visual analysis, the spiders evidently categorized both the blood-fed *Anopheles* stimulus and its disarranged version as their preferred prey. This suggests that they do not use a global, or holistic approach to recognition [4], [44], but rely instead on the analysis of specific elements at a local level to recognize an object [6]–[8]. This type of analysis functions much like distributed feature extraction algorithms of object recognition in computer vision based upon the vertebrate visual cortex [57], [58], in which low-level areas of the nervous system are delegated to recognizing different elements which are then fed to higher order centers [59]. A closer look at how these spiders visually analyze what it is they are seeing will provide a deeper understanding of what specific features these spiders are looking for when they are looking for prey.

## Methods

### a. General

All spiders used in this study were at least second generation laboratory reared individuals, and no juveniles tested had ever encountered mosquitos. Testing was carried between 0730 and 1200 h in a ÿharacteriz-controlled laboratory set to 24° with a photoperiod of 12L∶12D, lights on at 07∶00. Test spiders were unmated adults (body length, 4.5–5.5 mm) and juveniles (1.5–2.5 mm). Standard rearing and maintenance was as in earlier studies (for details, see [19], [20]). Spiders were caged individually and were fed to satiation once a week on *Drosophila* spp. Two h prior to their use as prey, *Drosophila* were given a honey and human blood (obtained from a blood bank) meal by inserting a cotton dental wick dipped in the mixture into their rearing container. Test spider hunger levels were standardized by a 5–7 day pretrial fast. Test spider predatory behaviors (noticing, stalking and/or pouncing) and their timing were recorded during all experiments. Noticing ÿharacte is ÿharacterized by the spider performing an optomotor response to face the stimulus with its AM eyes and subsequently staring continuously at the stimulus for a few seconds. Stalking behavior is ÿharacterized by the salticid slowly stepping toward the prey while visually fixated on the prey. Both are reliably identifiable behaviors.

### b. Stimuli

Stimuli consisted of videos of repeated sporadic movement of different images (Figure 1, Table 3), created using Adobe Photoshop CS5 in greyscale. Image 1 was a realistic line drawing of a blood-fed female *Anopheles gambiae* mosquito in typical resting posture, while the simplified images 2 (not blood-fed) and 3 (blood-fed) were similar but used only straight lines and ovals, with the latter depicting a blood-fed mosquito with an engorged abdomen, known as an important prey-identification cue [22]. Image 4 was a disarranged version of image 3, created so as to not alter the respective angles of any of the elements of image 3, while ensuring the elements were disconnected and, to humans, no longer resembling a mosquito. Image 5 was created by rendering a photograph of a housefly (*Musca domestica*) to grayscale and removing the background. Image 6, a circle the size of a housefly was created as a control, as were images 7 and 8. Image 7 was an altered version of image 4 where the angles of each of the elements of the image were altered and image 8 was a disarranged version of image 6, broken into 4 unequal sections. All images were created on a background of 250, 250, 250 RGB and had black pixel counts between ca. 200 and 550 pixels (Table 3). Screen size was set to 1024×768 pixels. All images were sized similarly and were presented at biologically relevant sizes (to the nearest 0.5 mm).

**Table 3:** Parameters of the images used in the stimuli of both experiments. Relative contrast is the number of black pixels in the frame.

Image	1	2	3	4	5	6	7	8
**Relative Contrast**	250	230	518	545	500	211	545	211
**Width (mm)**	7	6	6	6	7	6	7	7

For the actual images used see Figure 1.

To create the stimuli, one (in single-choice predatory behavior experiments) or two (in two-choice predatory behavior experiments) images were rendered into videos of repeated, horizontal (single-choice predatory behavior experiments) or vertical (two-choice predatory behavior experiments) motion (two bouts of back and forth movement every 10 s). Motion speed was 9°/s, at a viewing distance of 10 cm, and movement distance was set to be 8° visual angle. These parameters were selected to maximize the attention of the spiders [31], [32] (see Video S1 for a sample stimulus video).

Videos were projected onto a screen using an AAXA M2 Micro Projector connected to a computer, and placed 100 mm from the screen. The videos were played on a continuous loop using VLC player software. The screen was made of two protective sheets of glass (each 2 mm thick, 5 cm wide×5 cm long) with LCD screen polarizers from a Toshiba Tecra A9 PTS52C-MH409C laptop cut to size between them. This setup was used as we have found that the screen polarizers effectively reduce the brightness of the projected videos and did not result in a polarized image, while the glass sheets prevented the screen polarizers from getting damaged while being handled and cleaned. Due to the high spatial resolution of salticid principal eyes (ca. 11 minutes of arc, [25]) images projected directly onto a screen will appear pixelated once the spider gets close. To overcome this, while maintaining life size images at high resolution, larger than life size stimuli were back-projected through a lens placed between the projector and a screen, which reduced the projected image by ca. a factor of 10. Fine tuning the size of the projected stimuli was achieved by varying the size of the VLC player window on the computer monitor.

### c. Do Jumping Spiders View Abstract Images of Prey Elements as Prey?

**Figure 2:**
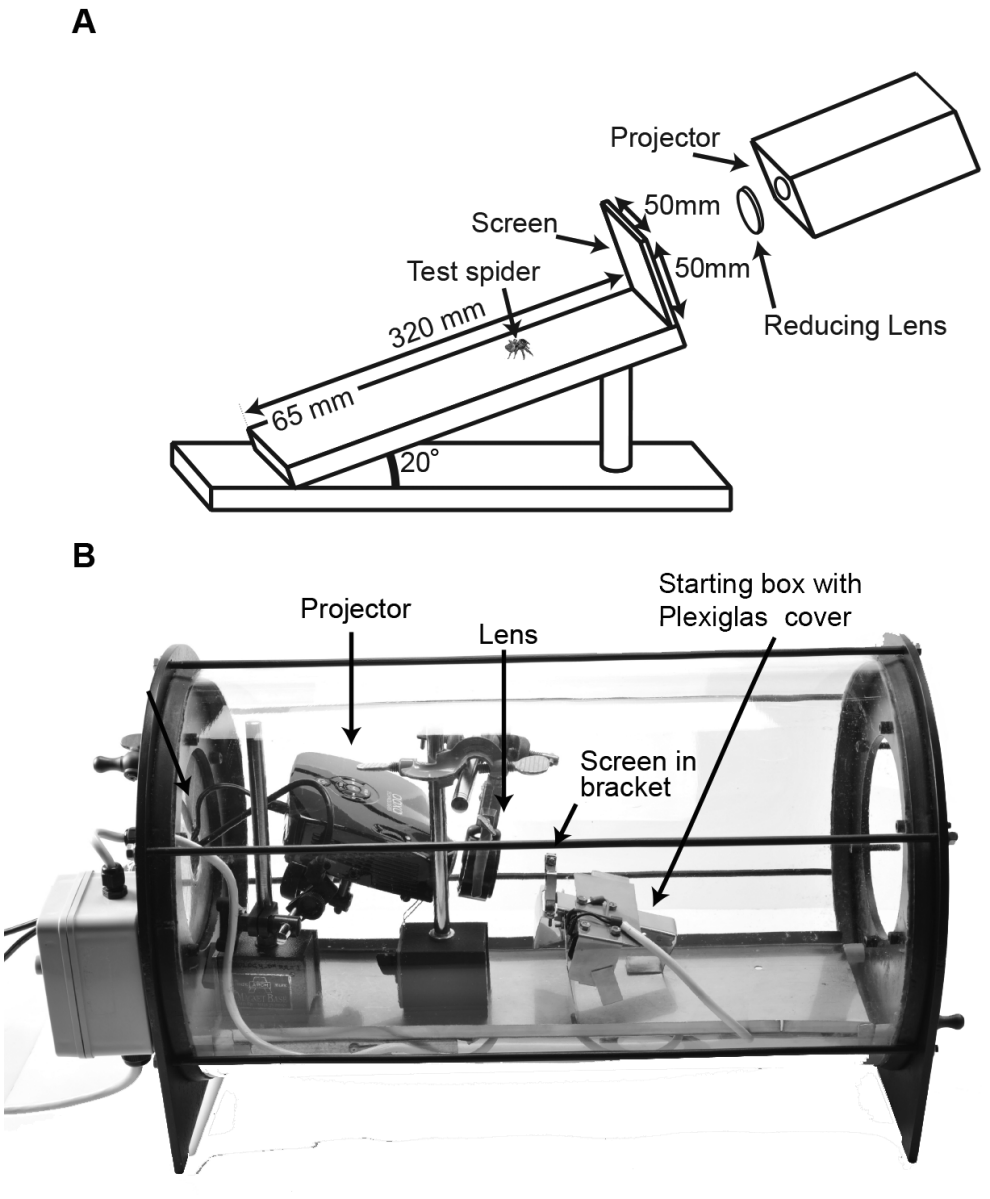
Experimental apparatuses used. a) Apparatus used in single-choice predatory behavior experiment. Spiders (not to scale) were placed either 10 cm (adults) or 6 cm (juveniles) away from stimulus screen, and behavior recorded. b) Apparatus used in the two-choice predatory behavior experiment. Projector and reducing lens placed inside glass chamber 100 mm from screen and ramp complex.

To answer this question, we tested the predatory responses of the spiders to individual stimuli (single-choice predatory behavior experiment). An angled wooden ramp supported by a wooden pole glued to a wooden base was placed in front of the screen and projector (see Figure 2a for dimensions). The apparatus was painted with two coats of polyurethane, but the top face of the ramp had a sticker marked with a 5 mm grid to allow accurate measurement of the spider’s distance from the stimulus when a particular behavior was observed. The ramp was wiped with 80% ethanol and allowed to dry for 15 min between each test to eliminate possible chemotactile traces from spiders in previous tests.

For each test, a spider was placed on the ramp and covered with a petri dish, at a distance of 6 (juveniles) or 10 (adults) cm from the center of the petri dish to the screen. These distances were used as they are far enough from the screen so that the spiders couldn’t ‘wald’ directly onto the stimuli, while being close enough to enhance the chances of the spiders reacting to the stimuli (juveniles were less responsive to stimuli at a distance compared to adults). The screen was covered with a piece of black cardboard until test spiders were released to prevent them seeing the stimulus until tests began. Once the spiders were relaxed (staying stationary or grooming) the screen was uncovered, the petri dish was removed and timing started. Tests ended when the spiders pounced on an image or walked/jumped off the ramp. If a spider noticed the image, the session was considered successful and tests were not repeated with the same spider. A spider that failed to notice the stimulus was tested up to twice in one day, or up to a total of 4 times in the following 3 days.

### d. Do Jumping Spiders View Abstract Images as their Preferred Prey?

In this experiment we relied on *E. culicivora’*s proven preference for *Anopheles* mosquitoes and presented them with a two-choice test. All spiders used in this test were laboratory reared and had no prior experience with mosquitoes. For these tests, rendered movies contained two images (Table 1) which moved identically and simultaneously. In each test, which image was on the right and which was on the left was randomized. The movies were projected as above, but experiments were held within a specialized apparatus containing a stainless steel ramp (15 mm wide×150 mm long; angled up by 25**°**) in front of the screen. The ramp was inside a glass chamber (diameter 300 mm, length 525 mm long) with removable sealing steel end plates (diameter 200 mm, kept off during this set of experiments). Welded to the ramp was a bracket onto which the screen was attached with a gap of 5 mm from the ramp. The ramp/screen unit (‘ramp complex’) sat mounted within holes on a stainless steel platform spanning the length of the cylinder (Figure 2b). In this way it could be removed for cleaning with 80% ethanol after each test and returned to the same place, while ensuring that the distance between the screen and the reducing lens and projector was always the same (and thus stimulus size was constant).

At a distance of 22 mm from the end of the ramp, a stainless steel ‘starting box’ (11 mm wide×19 mm high×22 mm deep; i.e., furthest point 44 mm from top end of ramp) was welded to the ramp complex (Figure 2b). The box had a transparent Plexiglas cover wired to an external controller so that it could be opened remotely. The spider was placed into the starting box and the door was closed. After 2 min, the ramp complex was put in place. Once the spider was away from the door of the starting box, after ca. 20 s, the door was opened and tests began. Tests ended with the spider pouncing on one of the two images on the screen or to jumping/walking off the ramp. Failing these two conditions, tests were stopped after 15 min. In this experiment we were interested in pouncing ÿehavior rather than instalking ÿehavior, as the former constitutes a more distinct choice by the spiders. For this reason, both adult and juvenile spiders were released a short distance from the screen (see Video S2 for a sample of the spider behavior in this experiment).

### e. Data Analysis

All analyses were done using SPSS Statistics v.20. For the single-choice predatory behavior experiment, GLM analyses were performed to check for the main effects of stimulus type, ‘sex’, relative contrast (number of black pixels against a white background) which was either in the ca. 200 or ca. 500 pixels) and their interaction on the spider’s choice to stalk the stimuli. Interactions between stimulus relative contrast and stimulus type were not analyzed, as these are nested. Sexes were divided into three – female, male and juvenile as their sex cannot be discerned and their behavior differs [22], [33]. In this model the dispersion parameter was set at 1, and type III sums of squares were used, though there was no qualitative difference from type I. Kruskal-Wallis tests were used to compare the predatory responses between the different sexes, with Mann-Whitney U tests for pairwise analysis. Cochran’s Q tests were used to test how the different stimuli affected the chances of the spiders noticing the stimulus and the propensity to stalk and pounce. Friedman tests were used to test the effects of the different stimuli on stalking initiation distance, as well as their effects on the amount of time it took the spiders to start stalking. When these effects were found to be significant, McNemar tests were used for pairwise comparisons. For the two-choice predatory behavior experiments, Binomial tests were used to test the spider’s choices, as well as possible side-bias.

## Supporting Information

Table S1 **Results from the single-choice predatory behavior experiment (all spiders).** M  =  Median, IQR  =  interquartile range. The percentages of the spiders that stalked/pounced are nested within the percent of spiders that noticed/stalked, respectively. See Figure 1 for stimulus images.
(DOC)

Table S2 **Statistics comparing between the different stimuli for the single-choice predatory behavior experiment (results from all spiders; data in Table S1).** *Cochran’s Q; **Friedman’s test (χ^2^); df = 6 in all tests.
(DOC)

Table S3 **Results from the single-choice predatory behavior experiment (female spiders).** M  =  Median, IQR  =  interquartile range. The percentages of the spiders that stalked/pounced are nested within the percent of spiders that noticed/stalked respectively. See Figure 1 for stimulus images.
(DOC)

Table S4 **Statistics comparing between the different stimuli for the single-choice predatory behavior experiment (results from female spiders; data in Table S3).** *Cochran’s Q; **Friedman’s test (χ^2^); df = 6 in all tests.
(DOC)

Table S5 **Results from the single-choice predatory behavior experiment (male spiders).** M  =  Median, IQR  =  interquartile range. The percentages of the spiders that stalked/pounced are nested within the percent of spiders that noticed/stalked, respectively. *Insufficient cases for IQR. See Figure 1 for stimulus images.
(DOC)

Table S6 **Statistics comparing between the different stimuli for the single-choice predatory behavior experiment (results from male spiders; data in Table S5).** *Cochran’s Q test; **Friedman’s test, χ2; ***Insufficient cases for analysis; in all tests, df = 6.
(DOC)

Table S7 **Results from the single-choice predatory behavior experiment (juvenile spiders).** M  =  Median, IQR  =  interquartile range. The percentages of the spiders that Stalked/Pounced are nested within the percent of spiders that Noticed/Stalked respectively. *Insufficient cases for IQR. **No juveniles tested with this stimulus. See Figure 1 for stimulus images.
(DOC)

Table S8 **Statistics comparing between the different stimuli for the single-choice predatory behavior experiment (results from juvenile spiders; data in Table S7).** *Cochran’s Q test; **Friedman’s test (χ^2^); in all tests, df = 5.
(DOC)

Table S9 **Results from the single-choice predatory behavior experiment for each sex/age group.** M  =  Median, IQR  =  interquartile range, F  =  female, M  =  male, Juv  =  juvenile. The percentages of the spiders that stalked/pounced are nested within the percent of spiders that noticed/stalked, respectively.
(DOC)

Table S10 **Statistics comparing between different sex/age groups for all stimuli in the single-choice predatory behavior experiment; data in Table S9.** Kruskal-Wallis tests (df = 2). In pairwise analysis females noticed the stimuli from significantly further away than other groups (Table S11).
(DOC)

Table S11 **Differences between the sex/age groups in notice distance, stalking propensity and pouncing propensity for all stimuli in the single-choice predatory behavior experiment (data in Table S9).** Pairwise analysis of differences in noticing distance, stalking propensities and pouncing propensities once stalking was initiated. Mann-Whitney U tests. F  =  female, M  =  male, J  =  juvenile.
(DOC)

Video S1 **Sample stimulus video for the two-choice predatory behavior experiment presenting images 3 and 4.**
(AVI)

Video S2 **Sample session video from the two-choice predatory behavior experiment.**
(MP4)
