# Supplementary material for: Innate Pattern Recognition and Categorization in a Jumping Spider
Source: PLoS One. 2014 Jun 3;9(6):e97819. doi: 10.1371/journal.pone.0097819 (PMC4043668; doi:10.1371/journal.pone.0097819)
Supplement: Table S1 — Results from the single-choice predatory behavior experiment (all spiders). M = Median, IQR = interquartile range. The percentages of the spiders that stalked/pounced are nested within the percent of spiders that noticed/stalked, respectively. See Figure 1 for stimulus images. (DOC) [file pone.0097819.s001.doc]

Table S1: Results from the single-choice predatory behavior experiment (all spiders).

| **Stimulus** | | **N** | | **% Noticed** | **Notice distance (cm)** | | **% Stalked** | | **Stalking initiation distance (cm)** | | **Decision time (s)** | **%  Pounced** | | |
| --- | --- | --- | --- | --- | --- | --- | --- | --- | --- | --- | --- | --- | --- | --- |
|  | |  | |  | **M/IQR** | |  | | **M/IQR** | | **M/IQR** | | |  |
| 1 | 33 | | 82 | | 6/5-7 | 74 | | 5.5/5-6.8 | | 5/2-24 | | | 90 | |
| 2 | 32 | | 88 | | 6/3.6-7 | 64 | | 5.75/4-7 | | 13/3-39 | | | 67 | |
| 3 | 30 | | 90 | | 6.5/5.5-7 | 74 | | 5.8/4.1-6.9 | | 10/4-21 | | | 75 | |
| 4 | 37 | | 81 | | 6.5/5.5-7.1 | 77 | | 6/5.5-6.5 | | 18/4-49 | | | 87 | |
| 5 | 32 | | 84 | | 5.5/4-7 | 56 | | 5/3.5-6 | | 14/2-32 | | | 73 | |
| 6 | 32 | | 84 | | 5.5/5-6.5 | 30 | | 5/3.1-6.5 | | 10/2-18 | | | 88 | |
| 7 | 40 | | 73 | | 8/5.3-9.8 | 17 | | 5/3.5-7 | | 8/4-10 | | | 80 | |

M = Median, IQR = interquartile range. The percentages of the spiders that stalked/pounced are nested within the percent of spiders that noticed/stalked, respectively. See Figure 1 for stimulus images.
