# Supplementary material for: Innate Pattern Recognition and Categorization in a Jumping Spider
Source: PLoS One. 2014 Jun 3;9(6):e97819. doi: 10.1371/journal.pone.0097819 (PMC4043668; doi:10.1371/journal.pone.0097819)
Supplement: Table S2 — Statistics comparing between the different stimuli for the single-choice predatory behavior experiment (results from all spiders; data in Table S1). *Cochran’s Q; **Friedman’s test (χ2); df = 6 in all tests. (DOC) [file pone.0097819.s002.doc]

Table S2: Statistics comparing between the different stimuli for the single-choice predatory behavior experiment (results from all spiders; data in Table S1).

|  | **Notice** | **Notice distance** | **Stalk** | **Stalking initiation distance** | **Decision time** | **Pounce** |
| --- | --- | --- | --- | --- | --- | --- |
| **Statistic** | *6.71 | **1.303 | *37.87 | **6.654 | **3.928 | *4.00 |
| **p** | = 0.349 | = 0.972 | < 0.001 | = 0.354 | = 0.686 | = 0.677 |

*Cochran’s Q; **Friedman’s test (χ^2^); df = 6 in all tests.
