# Supplementary material for: Innate Pattern Recognition and Categorization in a Jumping Spider
Source: PLoS One. 2014 Jun 3;9(6):e97819. doi: 10.1371/journal.pone.0097819 (PMC4043668; doi:10.1371/journal.pone.0097819)
Supplement: Table S3 — Results from the single-choice predatory behavior experiment (female spiders). M = Median, IQR = interquartile range. The percentages of the spiders that stalked/pounced are nested within the percent of spiders that noticed/stalked respectively. See Figure 1 for stimulus images. (DOC) [file pone.0097819.s003.doc]

Table S3: Results from the single-choice predatory behavior experiment (female spiders).

| **Stimulus** | **N** | **% Noticed** | **Notice distance (cm)** | **% Stalked** | **Stalking initiation distance (cm)** | **Decision time (s)** | **% Pounced** | |
| --- | --- | --- | --- | --- | --- | --- | --- | --- |
|  |  |  | **M/IQR** |  | **M/IQR** | **M/IQR** | |  |
| 1 | 11 | 82 | 6/4.8-7 | 67 | 5.5/4.8-6.6 | 13/2-25 | | 100 |
| 2 | 10 | 90 | 6/3.8-8 | 67 | 5.5/3.9-7.6 | 9/3-61 | | 67 |
| 3 | 9 | 100 | 7/6.8-8 | 67 | 6.75/5.1-7.8 | 15/8-156 | | 83 |
| 4 | 25 | 80 | 6.5/5.5-7.5 | 90 | 6/3.8-8.5 | 17/5-143 | | 89 |
| 5 | 9 | 100 | 6/3.5-7.3 | 56 | 4/2.8-8.5 | 14/11-29 | | 80 |
| 6 | 10 | 90 | 6.5/5.3-7.5 | 44 | 6/3.88-8.5 | 17/5-142 | | 100 |
| 7 | 23 | 87 | 8.8/6-10.8 | 15 | 5/2-5 | 9/8-9 | | 67 |

M = Median, IQR = interquartile range. The percentages of the spiders that stalked/pounced are nested within the percent of spiders that noticed/stalked respectively. See Figure 1 for stimulus images.
