# Supplementary material for: Innate Pattern Recognition and Categorization in a Jumping Spider
Source: PLoS One. 2014 Jun 3;9(6):e97819. doi: 10.1371/journal.pone.0097819 (PMC4043668; doi:10.1371/journal.pone.0097819)
Supplement: Table S4 — Statistics comparing between the different stimuli for the single-choice predatory behavior experiment (results from female spiders; data in Table S3). *Cochran’s Q; **Friedman’s test (χ2); df = 6 in all tests. (DOC) [file pone.0097819.s004.doc]

Table S4: Statistics comparing between the different stimuli for the single-choice predatory behavior experiment (results from female spiders; data in Table S3).

|  | **Notice** | **Notice distance** | **Stalk** | **Stalking initiation distance** | **Decision time** | **Pounce** |
| --- | --- | --- | --- | --- | --- | --- |
| **Statistic** | *6.857 | **4.409 | *14.195 | **4.233 | **2.747 | *5 |
| **p** | = 0.334 | = 0.621 | < 0.05 | = 0.645 | = 0.84 | = 0.544 |

*Cochran’s Q; **Friedman’s test (χ^2^); df = 6 in all tests.
