# Supplementary material for: Innate Pattern Recognition and Categorization in a Jumping Spider
Source: PLoS One. 2014 Jun 3;9(6):e97819. doi: 10.1371/journal.pone.0097819 (PMC4043668; doi:10.1371/journal.pone.0097819)
Supplement: Table S5 — Results from the single-choice predatory behavior experiment (male spiders). M = Median, IQR = interquartile range. The percentages of the spiders that stalked/pounced are nested within the percent of spiders that noticed/stalked, respectively. *Insufficient cases for IQR. See Figure 1 for stimulus images. (DOC) [file pone.0097819.s005.doc]

Table S5: Results from the single-choice predatory behavior experiment (male spiders).

| **Stimulus** | **N** | **% Noticed** | **Notice distance (cm)** | **% Stalked** | **Stalking initiation distance (cm)** | **Decision time (s)** | **% Pounced** |
| --- | --- | --- | --- | --- | --- | --- | --- |
|  |  |  | **M/IQR** |  | **M/IQR** | **M/IQR** |  |
| 1 | 8 | 75 | 5/2.4-7.5 | 50 | 5/* | 6/* | 67 |
| 2 | 8 | 88 | 3.5/3-6 | 43 | 5/* | 43/* | 33 |
| 3 | 9 | 78 | 6/4.5-6.5 | 57 | 4.5/3.8-5.6 | 8/1-39 | 25 |
| 4 | 9 | 89 | 6.5/5.6-7 | 50 | 5.75/5.5-6.8 | 31/8-39 | 75 |
| 5 | 8 | 88 | 5/3.5-6.5 | 29 | 5.5/* | 21/* | 50 |
| 6 | 9 | 67 | 5.5/3.6-7.6 | 17 | 2.5/* | 2/* | 0 |
| 7 | 17 | 53 | 6/2-8.8 | 22 | 5.5/* | 4/* | 50 |

M = Median, IQR = interquartile range. The percentages of the spiders that stalked/pounced are nested within the percent of spiders that noticed/stalked, respectively. *Insufficient cases for IQR. See Figure 1 for stimulus images.
