# Supplementary material for: Innate Pattern Recognition and Categorization in a Jumping Spider
Source: PLoS One. 2014 Jun 3;9(6):e97819. doi: 10.1371/journal.pone.0097819 (PMC4043668; doi:10.1371/journal.pone.0097819)
Supplement: Table S6 — Statistics comparing between the different stimuli for the single-choice predatory behavior experiment (results from male spiders; data in Table S5). *Cochran’s Q test; **Friedman’s test, χ2; ***Insufficient cases for analysis; in all tests, df = 6. (DOC) [file pone.0097819.s006.doc]

Table S6: Statistics comparing between the different stimuli for the single-choice predatory behavior experiment (results from male spiders; data in Table S5).

|  | **Notice** | **Notice distance** | **Stalk** | **Stalking initiation distance** | **Decision time** | **Pounce** |
| --- | --- | --- | --- | --- | --- | --- |
| **Statistic** | *3.778 | **11.095 | *5.636 | *** | *** | *** |
| **p** | = 0.707 | = 0.085 | = 0.465 | *** | *** | *** |

*Cochran’s Q test; **Friedman’s test, χ2; ***Insufficient cases for analysis; in all tests, df = 6.
