# Supplementary material for: Innate Pattern Recognition and Categorization in a Jumping Spider
Source: PLoS One. 2014 Jun 3;9(6):e97819. doi: 10.1371/journal.pone.0097819 (PMC4043668; doi:10.1371/journal.pone.0097819)
Supplement: Table S7 — Results from the single-choice predatory behavior experiment (juvenile spiders). M = Median, IQR = interquartile range. The percentages of the spiders that Stalked/Pounced are nested within the percent of spiders that Noticed/Stalked respectively. *Insufficient cases for IQR. **No juveniles tested with this stimulus. See Figure 1 for stimulus images. (DOC) [file pone.0097819.s007.doc]

Table S7: Results from the single-choice predatory behavior experiment (juvenile spiders).

| **Stimulus** | **N** | **% Noticed** | **Notice distance (cm)** | **% Stalked** | **Stalking initiation distance (cm)** | **Decision time (s)** | **% Pounced** |
| --- | --- | --- | --- | --- | --- | --- | --- |
|  |  |  | **M/IQR** |  | **M/IQR** | **M/IQR** |  |
| 1 | 14 | 86 | 6.3/5.1-7 | 92 | 5.5/5-7 | 4/3-11 | 91 |
| 2 | 14 | 86 | 6.5/4.5-7.4 | 75 | 7/4.5-7 | 8/4-26 | 78 |
| 3 | 12 | 92 | 6/4.5-7 | 91 | 5.5/4-6.6 | 7/5-15 | 90 |
| 4 | 3* | 67 | 3.75/* | 50 | 4/* | 334/* | 100 |
| 5 | 15 | 73 | 5/4-6.5 | 73 | 5/3.6-5.8 | 10/2-30 | 75 |
| 6 | 13 | 92 | 5.3/5-6 | 25 | 5/* | 4/* | 100 |
| 7 | ** | - | - | - | - | - | - |

M = Median, IQR = interquartile range. The percentages of the spiders that Stalked/Pounced are nested within the percent of spiders that Noticed/Stalked respectively. *Insufficient cases for IQR. **No juveniles tested with this stimulus. See Figure 1 for stimulus images.
