# Supplementary material for: Innate Pattern Recognition and Categorization in a Jumping Spider
Source: PLoS One. 2014 Jun 3;9(6):e97819. doi: 10.1371/journal.pone.0097819 (PMC4043668; doi:10.1371/journal.pone.0097819)
Supplement: Table S8 — Statistics comparing between the different stimuli for the single-choice predatory behavior experiment (results from juvenile spiders; data in Table S7). *Cochran’s Q test; **Friedman’s test (χ2); in all tests, df = 5. (DOC) [file pone.0097819.s008.doc]

Table S8: Statistics comparing between the different stimuli for the single-choice predatory behavior experiment (results from juvenile spiders; data in Table S7).

|  | **Notice** | **Notice distance** | **Stalk** | **Stalking initiation distance** | **Decision time** | **Pounce** |
| --- | --- | --- | --- | --- | --- | --- |
| **Statistic** | *2.545 | **6.101 | *14.261 | **4 | **7.467 | *4 |
| **p** | = 0.637 | = 0.192 | < 0.01 | = 0.406 | = 0.113 | = 0.406 |

*Cochran’s Q test; **Friedman’s test (χ^2^); in all tests, df = 5.
