# Supplementary material for: Innate Pattern Recognition and Categorization in a Jumping Spider
Source: PLoS One. 2014 Jun 3;9(6):e97819. doi: 10.1371/journal.pone.0097819 (PMC4043668; doi:10.1371/journal.pone.0097819)
Supplement: Table S9 — Results from the single-choice predatory behavior experiment for each sex/age group. M = Median, IQR = interquartile range, F = female, M = male, Juv = juvenile. The percentages of the spiders that stalked/pounced are nested within the percent of spiders that noticed/stalked, respectively. (DOC) [file pone.0097819.s009.doc]

Table S9: Results from the single-choice predatory behavior experiment for each sex/age group.

| **Sex/age** | **N** | **% Noticed** | **Notice distance (cm)** | **% Stalked** | **Stalking initiation distance (cm)** | **Decision time (s)** | **% Pounced** |
| --- | --- | --- | --- | --- | --- | --- | --- |
|  |  |  | **M/IQR** |  | **M/IQR** | **M/IQR** |  |
| F | 97 | 88 | 7/5.5-8 | 56 | 6/4.5-7 | 15/4-32 | 85 |
| M | 68 | 74 | 6/3.9-7 | 38 | 5/4.5-6 | 13/2-40 | 53 |
| Juv | 71 | 85 | 5.8/4.5-7 | 70 | 5/4-7 | 7/3-19 | 86 |

M = Median, IQR = interquartile range, F = female, M = male, Juv = juvenile. The percentages of the spiders that stalked/pounced are nested within the percent of spiders that noticed/stalked, respectively.
