# Supplementary material for: Innate Pattern Recognition and Categorization in a Jumping Spider
Source: PLoS One. 2014 Jun 3;9(6):e97819. doi: 10.1371/journal.pone.0097819 (PMC4043668; doi:10.1371/journal.pone.0097819)
Supplement: Table S10 — Statistics comparing between different sex/age groups for all stimuli in the single-choice predatory behavior experiment; data in Table S9. Kruskal-Wallis tests (df = 2). In pairwise analysis females noticed the stimuli from significantly further away than other groups (Table S11). (DOC) [file pone.0097819.s010.doc]

Table S10: Statistics comparing between different sex/age groups for all stimuli in the single-choice predatory behavior experiment; data in Table S9.

|  | **Notice** | **Notice distance** | **Stalk** | **Stalking initiation distance** | **Decision time** | **Pounce** |
| --- | --- | --- | --- | --- | --- | --- |
| **χ^2^** | 5.762 | 14.021 | 11.29 | 3.341 | 3.699 | 10.461 |
| **p** | = 0.056 | < 0.005 | < 0.005 | = 0.188 | = 0.157 | < 0.01 |

Kruskal-Wallis tests (df = 2). In pairwise analysis females noticed the stimuli from significantly further away than other groups (Table S11)
