# Supplementary material for: Innate Pattern Recognition and Categorization in a Jumping Spider
Source: PLoS One. 2014 Jun 3;9(6):e97819. doi: 10.1371/journal.pone.0097819 (PMC4043668; doi:10.1371/journal.pone.0097819)
Supplement: Table S11 — Differences between the sex/age groups in notice distance, stalking propensity and pouncing propensity for all stimuli in the single-choice predatory behavior experiment (data in Table S9). Pairwise analysis of differences in noticing distance, stalking propensities and pouncing propensities once stalking was initiated. Mann-Whitney U tests. F = female, M = male, J = juvenile. (DOC) [file pone.0097819.s011.doc]

Table S11: Differences between the sex/age groups in notice distance, stalking propensity and pouncing propensity for all stimuli in the single-choice predatory behavior experiment (data in Table S9)

|  | **Notice distance** | | | **Stalk** | | | **Pounce** | | |
| --- | --- | --- | --- | --- | --- | --- | --- | --- | --- |
|  | **F vs M** | **F vs J** | **M vs J** | **F vs M** | **F vs J** | **M vs J** | **F vs M** | **F vs J** | **M vs J** |
| **U** | 1471 | 1751 | 1486 | 1732.5 | 2205 | 1020 | -306.5 | 1005 | -267 |
| **p** | < 0.005 | < 0.005 | = 0.738 | < 0.05 | = 0.099 | < 0.001 | < 0.01 | = 0.968 | <0.01 |

Pairwise analysis of differences in noticing distance, stalking propensities and pouncing propensities once stalking was initiated. Mann-Whitney U tests. F = female, M = male, J = juvenile.
